# Active smoking is associated with severity of coronavirus disease 2019 (COVID-19): An update of a meta-analysis

**DOI:** 10.18332/tid/121915

**Published:** 2020-05-06

**Authors:** Fei R. Guo

**Affiliations:** 1 Department of Family Medicine, National Taiwan University Hospital, Taipei City, Taiwan; 2 Department of Family Medicine, College of Medicine, National Taiwan University, Taipei City, Taiwan

**Keywords:** smoking, COVID-19, meta-analysis

**Dear Editor,**

The letter to the Editor of Lippi and Henry^1^ published in the European Journal of Internal Medicine and entitled ‘Active smoking is not associated with severity of coronavirus disease 2019 (COVID-19)’ had errors and led to the wrong conclusion.

Lippi and Henry^1^ searched PubMed and Web of Science up to 9 March 2020, and identified 5 studies^2-2^ with data on smoking and severity of COVID-19. They performed a meta-analysis revealing a pooled OR of 1.69 (95% CI: 0.41–6.92) and concluded that active smoking does not seem to be significantly associated with enhanced risk of progressing towards severe disease in COVID-19. There were several mistakes in their data collection that led to errors in the meta-analysis. In table 1 of their letter, they indicated the outcome of Guan et al. study^2^ to be ‘Admission to ICU, mechanical ventilation, death’, however, they used the data of ‘severe disease’ in the study. According to the Guan et al.^2^ paper, the number of patients having composite outcome should be 66, and for patients not having a composite outcome should be 1019. However, Lippi and Henry^1^ used 172 and 913, respectively, in their paper. This is the most serious mistake because the Guan et al.^1^ study contributes to most of the cases in the meta-analysis. Moreover, the non-severe patients in the Huang et al.^3^ study should be 28 and not 31. The non-severe patients in the Yang et al.^5^ study should be 20 and not 18. The severe patients in the Zhang et al.^6^ should be 58 and not 60. The errors led to the wrong sample size of these 3 studies as well. Lippi and Henry^1^ were only correct in one^4^ out of the 5 studies.

**Figure 1:**
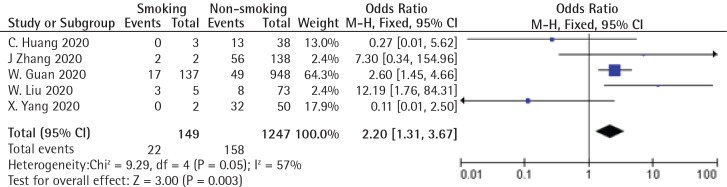
Forest plot of the updated meta-analysis

I performed an updated meta-analysis according to the correct data using RevMan Ver. 5.3, and provide the forest plot (Figure 1). The pooled OR was 2.20 (95% CI: 1.31–3.67; p=0.003). The heterogeneity was moderate (I>=57%). There was no obvious publication bias by the funnel plot. Though there are new studies published after the Lippi and Henry^1^ paper, the purpose of this letter is to correct their errors, therefore new studies are not included in the updated meta-analysis.

In a systemic review published by Vardavas and Nikitara^7^, 5 studies were included. Though meta-analysis was not performed in that study, the authors concluded that smoking is most likely associated with the negative progression and adverse outcomes of COVID-19. A recent meta-analysis including 7 studies also revealed that smokers have a double risk of severe COVID-19 (pooled OR=1.98; 95% CI: 1.29–3.05)^8^. A meta-analysis published in 2019 including 27 studies and 460592 participants revealed current smokers (pooled OR=2.17; 95% CI: 1.70–2.76) and ex-smokers (pooled OR=1.49; 95% CI: 1.26–1.75) were more likely to develop community-acquired pneumonia compared to never smokers^9^. The evidence suggests that smokers are more vulnerable to lung infection, and COVID-19 is no exception.

In conclusion, the results of this updated meta-analysis suggest that active smoking is significantly associated with the risk of severe COVID-19. Though more data are available now, they are not included in this study. However, the early meta-analysis of the Lippi and Henry^1^ paper should have had different results.
